# Bioactivity of Inositol Phosphates

**DOI:** 10.3390/molecules26165042

**Published:** 2021-08-20

**Authors:** Ivana Vucenik

**Affiliations:** 1 Department of Medical and Research Technology, University of Maryland School of Medicine, 100 Penn Street, Baltimore, MD 21201, USA; ivucenik@som.umaryland.edu; Tel.: +1-(410)-706-1832; Fax: +1-(410)-706-5229; 2 Department of Pathology, University of Maryland School of Medicine, Baltimore, MD 21201, USA

Inositol phosphates (IPs) are a huge and complex family of biomolecules, important in regulating vital cellular functions, signal transduction, energy transmission, and ion channels physiology and serving as structural components of cell membranes. Inositol hexaphosphate (IP_6_ or InsP_6_ or phytic acid) and its parent compound *myo*-inositol (Ins) are the most abundant, naturally occurring, and widely distributed among plants and mammals’ tissues.

In this special issue, a selection of research and review articles cover a wide range of bioactivity of inositol phosphates from occurrence to chemistry and methods for their determination, highlighting the broad spectrum of biological activities vital in health and diseases.

Reviewing specific signaling functions for IPs in different cell types, with new information regarding their cellular roles and cellular production, Maffucci and Falasca highlight the interaction of IPs with several proteins, protein complexes, and modulation of enzymatic activity, essential in regulation of several cellular processes [1], and discuss the possibility of new pharmacological opportunities and interventions that modulation of IPs levels can provide in multiple diseases, including cancer [1]. Inositol pyrophosphates (PP-IPs), such as 5-diphosphoinositol pentakisphosphate (5-IP_7_), are inositol metabolites containing high-energy phosphoanhydride bonds, involved in many key biological processes, including growth, vesicular remodeling, and energy homeostasis. The current understanding of how PP-IPs control mammalian cellular signaling networks in physiology and disease is summarized by Lee et al. [2]. IPs may also have distinctive properties in energy production, which is finely tuned to the metabolism and is known to be modulated by several signaling pathways, such as Akt/mTOR, in which IPs are key players [3]. Comparing plants and humans, Cridland and Gillaspy underline lessons we have learned from plants [4]. While eukaryotes have developed complex signaling pathways to adapt to a readily changing environment, in plants and humans, IPs have been implicated in phosphate and energy sensing. PP-IPs are synthesized from the phosphorylation of IP_6_, the most abundant IP. The plant PP-IP synthesis pathway is similar, but distinct from that of the human, reflecting differences in how molecules such as IP_3_ and IP_6_ function in plants vs. animals. In this review, they compare IP_6_ synthesis pathways, synthesis and regulation of the PP-IPs, and function of a specific protein domain called the Syg1, Pho1, Xpr1 (SPX) domain in binding PP-IPs and regulating inorganic phosphate sensing, providing novel insights into the biosynthetic pathway and bioactivity of these key signaling molecules in plant and human systems [4]. Of course, phosphorus (P) in plants is an essential nutrient, and IPs, particularly IP_6_, are important stores of P in plants.

Determination of IPs in biological fluids is difficult. From the early precipitation-based techniques, introduced more than a century ago, to the latest development of enzymatic bio- and nano-sensor applications, the analysis of IP_6_ and/or other IPs has never been a straightforward analytical task [5]. Due to the biomedical importance, several types of methodologies have been investigated over the years to develop a reliable determination of these intriguing analytes in many types of biological samples [5]. During the past 30 years, researchers have identified many important health benefits of IP_6_, inositol(s), and IPs. However, 150 years have elapsed since the discovery of IP_6_ to the first descriptions of its beneficial effects. This long delay may be due to the difficulty in determining phytate and other IPs in biological media, as discussed by Grases and Costa-Bauza [6], who dedicated a lot of time in developing methods for analyzing IPs in tissues and biological fluids.

Multiple beneficial effects on human health are related to IPs, and only few are highlighted here. Although IP_6_ is recognized as a strong antioxidant due to its specific configuration and properties of inhibiting hydroxyl radical formation, very few in vivo studies exist. Aguree et al. [7] examine the protective effect of IP_6_ in reducing the oxidative stress in a very specific animal model of human hereditary hemochromatosis, showing that IP_6_ protects against oxidative stress induced by genetic iron overload and high fat diets in β2-microglobulin knockout mice [7]. Blocking pathological calcifications is another property related to specific structure of IP_6_. It has been shown that IP_6_ and IPs have high capacity to inhibit calcium salt crystallization and that oral or topical administration of IP_6_ significantly decreases the development of pathological calcifications [6]. However, IP_6_ and Ins have recently received much attention for their roles in cancer prevention and treatment. A striking, consistent, and reproducible anticancer activity has been demonstrated in different experimental models and systems, targeting several key molecular targets, such as PI3K/Akt, mTOR, MAPK, PKC, and NF-κB. A few clinical studies have shown that IP_6_ alone or in combination with Ins is able to enhance the anticancer effect of conventional therapy, control cancer metastases, and improve quality of life. The historical perspectives, from the first experiments, concepts, and hypotheses to the first clinical observation in colon cancer patients is presented here [8], and that was the base of several clinical trials conducted later [9]. It is important that the prior research in the field is recognized and that credits are attributed properly. The potential of Ins and IPs in prevention and treatment of inflammatory bowel diseases, particularly colitis and colitis-induced carcinogenesis, was reviewed [10]. To better understand the molecular mechanisms of the anticancer action of IPs, Kapral et al. conducted the study on the involvement of microRNAs and showed that IP_6_ exerted its biological functions by downregulating the *miR-155* expression in human colon cancer cells [11]. However, the synergistic effect of IP_6_ and Ins was observed not only in cancer [8] but also in metabolic diseases. This combination has been beneficial in regulation of insulin secretion and was effective in the management of type 2 diabetes mellitus [12]. So, inositol(s) may also have distinctive properties in energy metabolism and metabolic disorders [3,13]. Targeting the inositol pyrophosphate biosynthetic enzymes in metabolic diseases is a possible therapeutic approach [13]. The IP_6_K pathway is a potential target in obesity and other metabolic diseases [13]. Furthermore, IP_6_K isoforms have been proposed as possible targets for cancer therapy [14]. That is not surprising, because both insulin resistance and cancer share several perturbed but critical biochemical pathways, and interestingly both are affected by inositol. Moreover, because of the role of insulin signaling and hormonal synthesis in the ovaries, and involvement of Ins in a number of biochemical pathways within oocytes, the beneficial effect of Ins, D-chiro-inositol, and their combination was shown in polycystic ovarian syndrome (PCOS) by improving the metabolic, hormonal, and reproductive aspects of PCOS [15].

In conclusion, there has been fascination for plants stretching back to millennia, and humans have remained dependent on plants for medicines. The plant kingdom is rich with organic compounds for traditional medicine, as well as for the development of novel agents to improve human health, as was shown for Ins, IP_6_, and IPs.
